# Cholesteryl ester transfer protein inhibition is associated with reduced risk of Sjögren’s syndrome

**DOI:** 10.1093/rheumatology/kead115

**Published:** 2023-03-10

**Authors:** Sizheng Steven Zhao, Sarah Dyball

**Affiliations:** Centre for Epidemiology Versus Arthritis, Division of Musculoskeletal and Dermatological Science, School of Biological Sciences, Faculty of Biological Medicine and Health, The University of Manchester, Manchester Academic Health Science Centre, Manchester, UK; Centre for Epidemiology Versus Arthritis, Division of Musculoskeletal and Dermatological Science, School of Biological Sciences, Faculty of Biological Medicine and Health, The University of Manchester, Manchester Academic Health Science Centre, Manchester, UK

Rheumatology key message

- Genetic evidence supports CET P ihibition as a potential therapeutic target for reducing risk of Sjögren’s syndrome.

Dear Editor, Sjögren’s syndrome (SS) is a chronic, systemic autoimmune disease primarily characterized by lymphocytic infiltration of exocrine glands, but also extraglandular manifestations such as vasculitis, inflammatory arthritis, and pulmonary and neurological dysfunction. Management is typically symptomatic. No disease-modifying drugs have been approved for SS to date. Placebo-controlled trials of systemic therapies, such as rituximab and abatacept, have largely been negative [1]. Thus there is urgent need to identify and develop disease-modifying agents.

Lipid profiles differ between SS cases and healthy controls [2], but their relevance for patho-aetiology is unclear. Cholesteryl ester transfer protein (CETP), a drug target for cholesterol reduction and coronary heart disease, has been implicated in the pathogenesis of a related autoimmune disease, SLE (unpublished data). We used large population-level data to examine the association between genetically proxied CETP inhibition (CETPi) and the risk of SS.

Natural variation in the gene that encodes a protein target can offer insights into the clinical effects of perturbing the target pharmacologically. In this two-sample mendelian randomization (MR) analysis, we proxied CETPi using single nucleotide polymorphisms (SNPs) within or near the *CETP* gene (build GRCh37/hg19: chromosome 16: 56995762–57017757) ± 100 kb that are minimally correlated (*r*^2^ < 0.1) and associated with circulating low-density lipoprotein cholesterol (LDL) levels at genome-wide significance (*P* < 5 × 10^−8^) from a genome-wide association study (GWAS) of ∽1.3 million European individuals [3]. LDL was chosen as the biomarker because CETPi was effective for LDL reduction in clinical trials. To examine whether potential causal effects act exclusively through CETP or LDL lowering overall, we also measured LDL cholesterol using variants across the genome (*r*^2^ < 0.001, *P* < 5 × 10^−8^).

Genetic association data for primary SS was taken from the largest GWAS to date of 6098 patients, defined by American-European Consensus Group criteria, and 34 928 controls [4]. Causal estimates were derived using the ratio method and pooled using the inverse variance weighted method. We accounted for the weak correlation of CETP variants using their linkage disequilibrium matrix. MR estimates were scaled to a 1 s.d. (i.e. ∽6.7 mmol/mol) reduction in LDL. MR sensitivity analyses were performed to examine potential bias from pleiotropy, and colocalization was used to examine potential genetic confounding through linkage disequilibrium.

Ten SNPs were selected to proxy CETPi (Supplementary Table S1, available at *Rheumatology* online). Genetically proxied CETPi was associated with a reduced risk of SS [odds ratio (OR) 0.17 per s.d. reduction in LDL (95% CI 0.08, 0.36); *P* = 0.02]. Estimates were directionally concordant in pleiotropy robust sensitivity analyses (Supplementary Table S2 and Fig. S1). The probability of colocalization conditional on the presence of a causal variant for the outcome was 96%, i.e. the results were unlikely attributable to genetic confounding (Supplementary Table S3). A genetically proxied reduction in LDL overall was not associated with SS risk [OR 0.96 (95% CI 0.78, 1.20); *P* = 0.74].

These results provide supportive genetic evidence for CETP as a therapeutic target for reducing the risk of SS. To our knowledge, this is the first study to implicate CETP in SS patho-aetiology. CETP is produced in the liver and adipose tissue and promotes the transfer of cholesteryl esters from HDL into triglyceride-rich lipoproteins [5]. CETP inhibitors were developed for cardiovascular disease, with conflicting efficacy. Anacetrapib, but not other CETP inhibitors, reduced non-HDL cholesterol and major coronary events with a good safety profile [6]. The precise role of CETP in SS is unclear, but appears to be independent of its effect on LDL levels.

Key limitations of any MR analysis are the assumptions of no confounding or pleiotropic pathways. The functionality of selected variants is incompletely understood, thus pleiotropy remains possible. However, sensitivity analyses were consistent with the main results. In summary, this study supports CETP as a therapeutic target for reducing the risk of SS, but further mechanistic studies are needed to confirm our findings.

## Supplementary Material

kead115_Supplementary_Data

## Data Availability

The genome-wide association summary statistics for SS can be obtained from the Databases of Genotypes and Phenotypes under accession number phs002723.v1.p1. Summary data for LDL cholesterol can be obtained from http://csg.sph.umich.edu/willer/public/glgc-lipids2021.
