# Supplementary material for: Cholesteryl ester transfer protein inhibition is associated with reduced risk of Sjögren’s syndrome
Source: Rheumatology (Oxford). 2023 Mar 10;62(9):e258–9. doi: 10.1093/rheumatology/kead115 (PMC10473269; doi:10.1093/rheumatology/kead115)
Supplement: kead115_Supplementary_Data [file kead115_supplementary_data.docx]

Supplementary Materials for:

**Cholesteryl ester transfer protein (CETP) inhibition is associated with reduced risk of Sjogren’s disease**

Sizheng Steven Zhao^1^, Sarah Dyball^2^

1 Centre for Epidemiology Versus Arthritis, Division of Musculoskeletal and Dermatological Science, School of Biological Sciences, Faculty of Biological Medicine and Health, The University of Manchester, Manchester Academic Health Science Centre, Manchester, UK.

Correspondence to: Sizheng S Zhao. Centre for Epidemiology Versus Arthritis, Division of Musculoskeletal and Dermatological Science, School of Biological Sciences, Faculty of Biological Medicine and Health, The University of Manchester, Manchester Academic Health Science Centre, Oxford Road, Manchester, M13 9LJ, UK. Email: Sizheng.zhao@manchester.ac.uk

Contents

[**Methods** 2](#_Toc129166238)

[**Table S1. Variants used to instrument CETP.** 3](#_Toc129166239)

[**Table S2. Results of primary and pleiotropy robust sensitivity analyses.** 3](#_Toc129166240)

[**Table S3. Colocalization results for CETP and Sjögren’s disease.** 4](#_Toc129166241)

[**Figure S1. Scatter plot for analysis of CETP and Sjögren’s disease.** 5](#_Toc129166242)

[**References** 6](#_Toc129166243)

### **Methods**

A detailed description of the drug target mendelian randomisation (MR) and colocalization methods used herein are available from supplementary materials of reference [1].

Briefly, MR is an analytical method that provides evidence about causal relationships between modifiable exposures and disease outcomes, using genetic variants that are associated with exposure variation at a population level [2–4].

A genetic variant can be considered as a valid instrumental variable for an exposure if it satisfies the instrumental variable assumptions: it is associated with the exposure in a specific way that does not affect the outcome except via the exposure, and it is not associated with the outcome due to confounding. The genetic variant is not required to be causal for the exposure to meet these assumptions, but causal variants are more likely to fulfil the assumptions.

To complement MR analyses, we used colocalization to test the probability that LDL levels and Sjogren’s disease have a shared causal variant at the *CETP* gene. We used the *coloc* R package [5] which implements Bayesian colocalization assuming one shared variant and that the causal variant is included in the GWAS. Outputs include the posterior probability of distinct causal variants (H3) and shared causal variant (H4). The primary output of interest was the relative probabilities of H3 and H4, where H4/(H4+H3) represents the probability of colocalization conditional on the presence of a causal variant for the outcome [6].

We sought for potentially pleiotropic variants using PhenoScanner [7] – a genotype-phenotype database –for associations with other (e.g., immune) traits that may represent pleiotropic pathways. There were no variants associated with such traits at p<1e-5 (adjusted for multiple testing).

### **Table S1. Variants used to instrument CETP.**

| SNP | Chromosome | Position | Effect allele | Other allele | EAF | Beta | SE | P-value |
| --- | --- | --- | --- | --- | --- | --- | --- | --- |
| rs9924336 | 16 | 56908703 | T | C | 0.191 | -0.01006 | 0.001783 | 1.68E-08 |
| rs9921780 | 16 | 56952098 | G | A | 0.427 | 0.008706 | 0.001408 | 6.21E-10 |
| rs72786781 | 16 | 56970210 | A | T | 0.0261 | -0.0271 | 0.004625 | 4.64E-09 |
| rs247617 | 16 | 56990716 | A | C | 0.321 | -0.03667 | 0.001482 | 3.14E-135 |
| rs6499863 | 16 | 56992017 | A | G | 0.16 | 0.013986 | 0.001886 | 1.21E-13 |
| rs7203984 | 16 | 56999258 | C | A | 0.193 | 0.026992 | 0.001761 | 5.10E-53 |
| rs289718 | 16 | 57009932 | C | T | 0.311 | -0.01192 | 0.001509 | 2.72E-15 |
| rs56208677 | 16 | 57010232 | T | C | 0.0677 | -0.02419 | 0.002787 | 3.98E-18 |
| rs12708983 | 16 | 57014411 | C | T | 0.0362 | -0.02454 | 0.003856 | 1.97E-10 |
| rs1800777 | 16 | 57017319 | A | G | 0.0356 | 0.022113 | 0.003736 | 3.25E-09 |
| rs12720917 | 16 | 57019392 | C | T | 0.147 | -0.01174 | 0.002063 | 1.25E-08 |

### **Table S2. Results of primary and pleiotropy robust sensitivity analyses.**

| Exposure | Method | No. SNPs | OR | Lower 95%CI | Upper 95%CI | P-value |  |
| --- | --- | --- | --- | --- | --- | --- | --- |
| **CETP** | **Inverse variance weighted*** | **10** | **0.17** | **0.08** | **0.36** | **0.02** |  |
| CETP | MR Egger | 10 | 0.08 | 0.01 | 0.92 | 0.08 |  |
| CETP | Weighted median | 10 | 0.16 | 0.04 | 0.64 | 0.01 |  |
| **CETP** | **Weighted mode** | **10** | **0.17** | **0.04** | **0.75** | **0.04** |  |
| LDL | Inverse variance weighted | 314 | 0.96 | 0.78 | 1.20 | 0.74 |  |
| LDL | MR Egger | 314 | 1.09 | 0.76 | 1.56 | 0.65 |  |
| LDL | Weighted median | 314 | 0.95 | 0.71 | 1.25 | 0.70 |  |
| LDL | Weighted mode | 314 | 0.99 | 0.73 | 1.33 | 0.93 |  |
| *Additionally accounting for minimal correlation between CETP instruments. | | | | | | | |

### **Table S3. Colocalization results for CETP and Sjögren’s disease.**

| H0 | H1 | H2 | H3 | H4 | H4/(H4+H3) |
| --- | --- | --- | --- | --- | --- |
| 4.16E-128 | 8.72E-01 | 2.49E-130 | 5.10E-03 | 1.23E-01 | 9.60E-01 |
| Posterior probability for  H0: neither trait has a genetic association in the region;  H1: only trait 1 has a genetic association in the region;  H2: only trait 2 has a genetic association in the region;  H3: both traits are associated, but with different causal variants;  H4: both traits are associated and share a single causal variant.  H4/(H3+H4) represents the probability of colocalization conditional on the presence of a causal variant for the outcome | | | | | |

### **Figure S1. Scatter plot for analysis of CETP and Sjögren’s disease.**


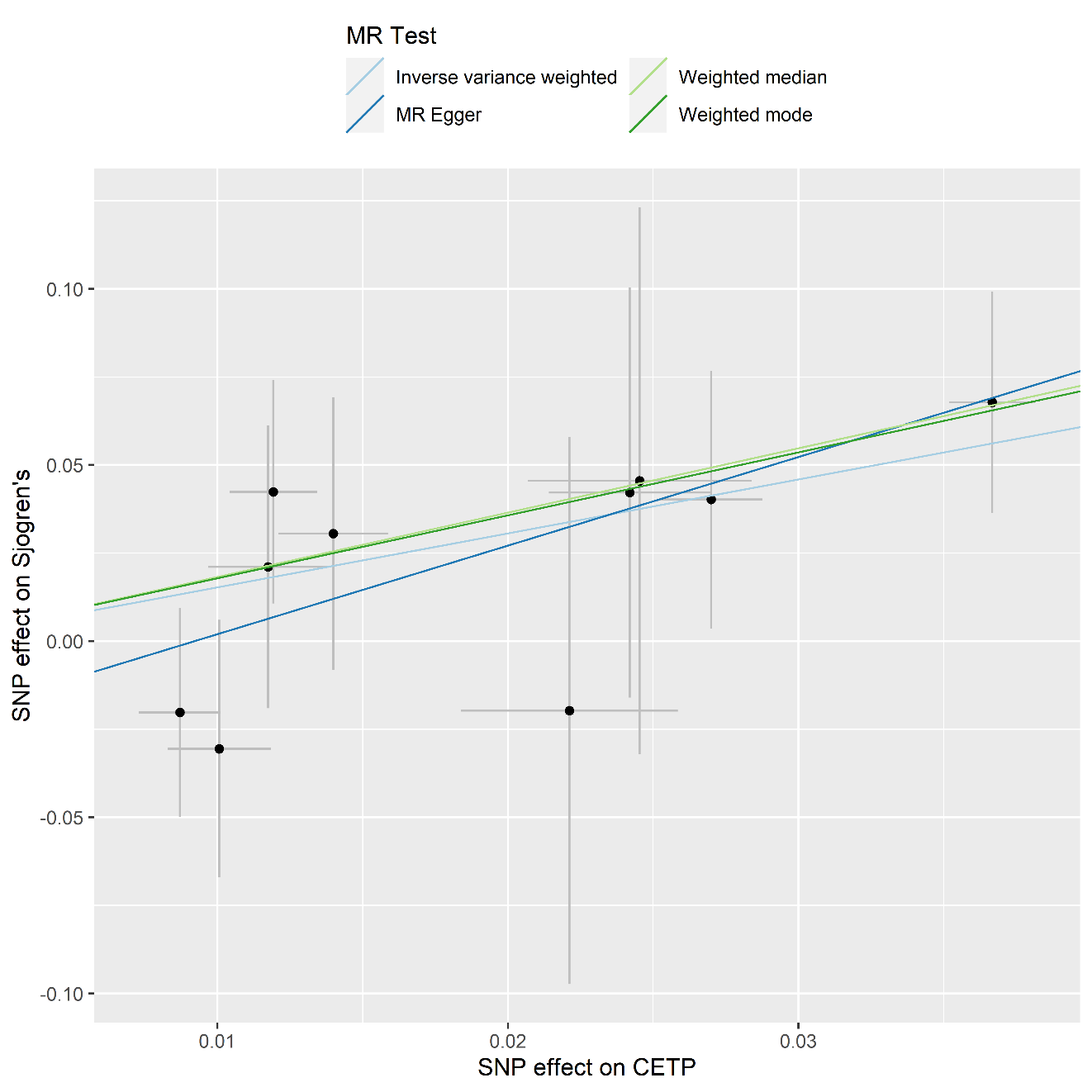


### **References**

1. Zhao SS, Karhunen V, Morris AP, Gill D. ADAMTS5 as a therapeutic target for osteoarthritis: Mendelian randomisation study. Ann Rheum Dis 2022;81:903–4.

2. Smith GD, Ebrahim S. ‘Mendelian randomization’: can genetic epidemiology contribute to understanding environmental determinants of disease? International Journal of Epidemiology 2003;32:1–22.

3. Davies NM, Holmes MV, Smith GD. Reading Mendelian randomisation studies: a guide, glossary, and checklist for clinicians. BMJ [Internet] 2018 [cited 2020 Jul 30];362. Available from: https://www.bmj.com/content/362/bmj.k601

4. Gill D, Georgakis MK, Walker VM, Schmidt AF, Gkatzionis A, Freitag DF, et al. Mendelian randomization for studying the effects of perturbing drug targets. Wellcome Open Res 2021;6:16.

5. Colocalisation Tests of Two Genetic Traits [Internet]. [cited 2021 Dec 10];Available from: https://chr1swallace.github.io/coloc/

6. Zuber V, Grinberg NF, Gill D, Manipur I, Slob EAW, Patel A, et al. Combining evidence from Mendelian randomization and colocalization: Review and comparison of approaches. Am J Hum Genet 2022;109:767–82.

7. Staley JR, Blackshaw J, Kamat MA, Ellis S, Surendran P, Sun BB, et al. PhenoScanner: a database of human genotype-phenotype associations. Bioinformatics 2016;32:3207–9.
